# Utilising an Allied Health Practitioner Capability Audit and Confidence Survey to Identify Implications for Telehealth Safety and Risk—A Chronicle of a Health Service Improvement Activity

**DOI:** 10.3390/healthcare12141442

**Published:** 2024-07-19

**Authors:** Raeleen Parker, Hayley Gough, E-Liisa Laakso

**Affiliations:** 1 Allied Health Service Development, Mater Health, South Brisbane, QLD 4101, Australia; rae.parker@health.qld.gov.au; 2 Consumer Partnering, Metro South Hospital and Health Service, Eight Miles Plains, QLD 4113, Australia; 3 Mater Education, South Brisbane, QLD 4101, Australia; 4 Mater Research Institute, University of Queensland, South Brisbane, QLD 4101, Australia

**Keywords:** telehealth, technology, allied health, allied health professional, competence, confidence, co-design

## Abstract

Whilst the benefits of telehealth were identified during the COVID-19 pandemic, we noted barriers to its use at a vital time. Through a health service improvement approach, we sought to increase allied health professional capability in telehealth, but we also sought to understand if there were risks associated with its use. We designed and implemented tools to evaluate allied health professional competence and confidence in using telehealth with private and public patients in a metropolitan teaching hospital setting. With an emphasis on technology capability, we undertook audits over three consecutive years (2020 to 2022) of allied health professional telehealth occasions of service reporting on compliance with the audit criteria and investigating staff confidence in undertaking telehealth sessions using a co-designed survey. The audit tool and confidence survey results were used to identify risk factors to telehealth service delivery using a Modified Health Failure Modes, Effects Analysis. Although confidence levels were relatively high among staff, confidence in managing safety factors and technology risks associated with telehealth were not initially verified by the audit findings. Remedial efforts resulted in service improvements in many identified risk factors, yet technology performance and its troubleshooting remained a primary variable in the ability of staff to comply with the requirements of the real-time audits. Health workers using telehealth should have training to engage safely and effectively in telehealth care and the technology.

## 1. Introduction

Contemporary health care increasingly utilises digital health practices as part of virtual care. Butler-Henderson et al. [1] contend that digital health will drive health reform, and the allied health (AH) workforce needs to be digital-health-ready with the right capabilities. One facet of digital health is synchronous, internet-facilitated telehealth (TH), which was used extensively during the COVID-19 pandemic. Yet, until COVID-19, there had been a paucity of literature regarding factors relating to AH TH capability and associated risks. Only recently have others identified service components and strategies for sustaining AH TH [2].

In the context of AH staff TH capability, since 2017, we have been interested in assessing the TH practice and confidence of the allied health professional (AHP) workforce to determine what is required for offering high-quality and low-risk TH services. We have previously reported on the staff and consumer views of TH noting that patients preferred a mix of TH and face-to-face appointments, and staff experienced technology barriers and challenges to providing high-quality TH [3]. We have reported three functional tiers needing consideration when developing high-quality AHP TH capability: organisational, departmental and individual factors (AHP and patient). In general, health practitioner capability includes a combination of skills, knowledge, values and self-esteem, all of which should be supportive of AH staff to capably use TH [4]. However, the diverse mix of factors that can contribute to AH TH capability has meant the need to develop and implement ways by which to understand and identify factors that potentially contribute to risks associated with the use of TH. By understanding capability and thereby risks, we expected that we could optimise the use of TH by qualified AHPs in a metropolitan teaching hospital. The target patient population for the use of TH is metropolitan community dwellers who access TH at work or home.

To assess AH TH capability and in the absence of a suitable tool, we recognised the need to develop an audit tool. To select which criteria to audit, in early 2020, we turned to the literature, which offered four factors necessary for high-quality TH provision including technology, usability, physical environment and human attributes [5]. Brunner and colleagues [6] identified four eHealth capability domains (digital technologies, systems, policies; clinical practice and applications; data analysis/knowledge creation; and system/technology implementation) that could be expected of workforce-ready health practitioners. These factors broadly informed our thoughts on what information was necessary for auditing TH practice. 

We also undertook a search of internal hospital and external policies and evidence-based standards relevant to TH safety, efficiency, quality, experience and sustainability, using search terms including the hospital name, “allied health” (including professions typically defined as allied health, such as physiotherapy, occupational therapy, etc.) and “telehealth”. The search for Australian AH competency standards identified significant gaps regarding the inclusion of digital health competencies across professional standards, with no best practice guide from which AH professions might commence the development of TH capabilities [1]. There were limited Australian health professional organisations [7,8,9,10,11] with published TH practice guidelines, although the COVID-19 pandemic would eventually determine the need for contemporising competency standards for digital health. Considerate of the literature and document findings, audit tool criteria were selected to represent quality and safety, technology and logistical and operational matters related to delivering effective TH.

As well as operational and organisational compliance requirements which could be measured by audit, TH capability requires an understanding of staff values and attitudes. In our case, we wanted to know more about AHP confidence in their use of TH. Perspectives of TH confidence by AHPs have received some attention. Cottrell et al. [12] reported survey outcomes expressed by multidisciplinary team members offering a home-based TH service to people with spinal pain. The authors noted that “…despite a high level of confidence in the general use of computers, clinicians initially demonstrated limited confidence and knowledge in the use of telerehabilitation…”, but confidence, acceptance and satisfaction increased with training, experience and appropriate technologies. To understand AHP TH capability that was specific to an acute hospital setting, we sought to interrogate ratings of confidence by AH staff when using TH as a health care modality. As the survey instruments available at the time [13] were not suitable for our needs, we set out to design a tool fit for the purpose of improving the quality of the AH TH service. 

The purpose of our work was centred on measuring the capability (confidence and competence) of AH staff to deliver a high-quality, hospital-based TH service and seeing if capability factors posed risks to patients, staff or the organisation. In this paper, we report on the health service improvement approach we undertook over three years (2020 to 2022) to advance AHP TH capability using a co-designed audit tool (for observing real-time, synchronous TH sessions and the audit of associated documents) and a confidence survey. Using the results of the audits and surveys, we aimed to determine if there were risks associated with the reported levels of confidence and competence in the use of TH by AHPs. If we identified risks, we also wanted to know what those risks might be and to develop recommendations for service improvement and risk reduction. 

## 2. Methods

### 2.1. Project Design

**Figure 1:**
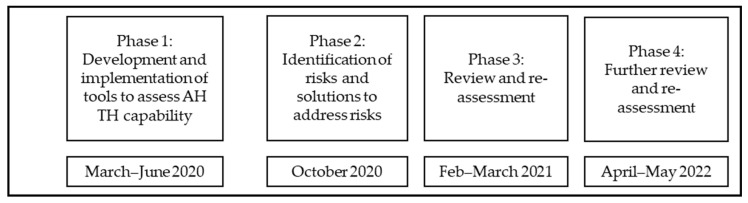
Design of health service improvement activities (AH: allied health; TH: telehealth).

This was a service improvement activity using mixed methods, designed to identify and assess risks associated with AH TH service delivery (Figure 1). To identify risks, our initial objectives were to understand if (1) the organisational TH requirements were being achieved when delivering AH services; (2) AHP staff were confident to meet AH TH organisational and clinical requirements; and (3) the TH audit tool and staff confidence survey assisted in improving TH service delivery and mitigating any identified risks. The project design included the development and use of an observational audit of real-time TH and related documents and a staff confidence survey enacted initially during the peak of the SARS-CoV-2 pandemic in 2020 with follow-up audits and surveys in 2021 and 2022. 

All key stakeholders were briefed on the concepts of TH confidence and competence and the methodology and structure of the TH audit and staff surveys. Directors and managers of relevant clinical areas were notified of the audit and survey through email, phone conversations and face-to-face meetings. The work reported herein was considered exempt from a human research ethics review (EXMT/MML/82092 (V2)).

Under the guidance of the hospital education unit and the AH Research Fellow, a Community of Practice (CoP) consisting of AH department TH champions was involved in the development of methods to assess AHP competence and confidence in using TH [3]. In addition to the first two project objectives, the CoP identified several subsidiary questions to be incorporated into the measures to aid in identifying risks (Table 1).

### 2.2. Phase One—Development and Implementation of Tools

#### 2.2.1. Allied Health Professional TH Audit Tool

Based on the findings of our initial literature search, a capability audit tool was constructed in 2020 to address our first two objectives (and the subsidiary questions) and to investigate factors related to consumer standards (including a document and system review), preparation for the TH appointment (with sub-sections including quality, privacy, patient risk, technology and clinical care), clinical care from the AHP perspective and consumer management of the TH environment. The content of the finalised audit tool is available in Table A1. 

The audit tool was reviewed by the authors, members of the CoP and members of the hospital’s Allied Health Management Committee. The audit elements were entered into the hospital measurement, analysis and reporting system (MARS; https://www.opus5k.com, Australia, URL accessed 27 June 2024) and tested and refined by two of the authors (RP and HG) and the multidisciplinary CoP members during simulated patient TH sessions. This process assured the audit tool was suitable across AH professions and demonstrated auditor agreement using, predominantly, ratings of Yes, No, NA (“Not Applicable”). The process was designed to increase the likelihood of being able to observe and report TH behaviours during an audit session. As technology issues had been noted as specific capability issues during our earlier work, the audits included a focus on such factors. 

To standardise the audits, a procedure document was developed and included a call for AHP volunteers to be audited. Staff who volunteered for the audits were briefed, and consents to observe TH interactions were obtained from the AHPs and the patients whose TH appointment was to be audited. The initial audits were conducted by two of the authors (RP and HG) from April to May 2020, and results were recorded in MARS. 

As our third objective sought to evaluate whether there were any improvements in the provision of AH TH service, after the initial implementation of the audit tool in April–May 2020, the tool was reviewed, and minor modifications were made in February 2021 by the multidisciplinary CoP members. Modifications were made to ensure the audit tool reflected newly published guidelines [14] from professional associations and internal changes implemented after the initial audit (e.g., workstation ergonomics). Space was added within the tool to allow auditors to note additional comments and observations. For subsequent audits in 2021 and 2022, the CoP members volunteered as auditors. To maintain a standardised approach from year to year, the audit procedure was reviewed, and an audit simulation activity was designed and completed by the CoP members. Each auditor agreed to complete at least two TH sessions in their own AH department and two TH sessions in another AH department. Only AH staff who used TH were eligible to be audited.

#### 2.2.2. Allied Health Professional TH Confidence Survey

The AHP TH confidence survey was developed to assist in understanding the staff perspective in mapping the effects of ongoing TH quality improvement measures. The primary factors considered were staff perceptions regarding hospital organisational support for TH, an emphasis on AH staff confidence to use TH technology and their confidence to use TH clinically to achieve patient outcomes. The anonymous survey was designed iteratively to ensure it could be completed quickly and efficiently by busy AHPs. The questions were modelled on an earlier internal TH survey used by AH staff working in a community setting. The confidence survey contained questions designed to triangulate findings from the audit, e.g., information regarding patient choice to continue using TH in ongoing care. A mix of demographic, quantitative (closed questions) and qualitative open-ended questions were included in the survey to obtain a broad overview of factors deemed relevant to staff confidence (Table A2). Survey respondents were asked to rate confidence on a 5-point scale (“Very confident”, “Fairly confident”, “Neither”, “Not very confident”, “Not at all confident”). The questions were tested and evaluated by members of the CoP and modified to aid clarity. The confidence survey link was sent via internal email to all hospital AH staff and open for a one-week period in June 2020, using a cloud-based survey instrument (http://www.surveymonkey.com, URL accessed 26 June 2024). Directors of AH promoted survey completion to staff. If staff were not users of TH, they were not eligible to complete the survey.

After the initial round of surveys in May–June 2020, the confidence survey was reviewed, and the survey method was repeated annually in 2021 and 2022 with modifications to some survey questions to reflect responses from each previous survey round. Due to the modifications, not all questions were able to be compared from year to year. 

#### 2.2.3. Impact and Severity Risk Matrix

In 2020 and 2021, results from the audits and confidence survey were evaluated with an Impact and Severity Risk Rating Matrix using the modified Health Failure Modes, Effects Analysis (HFMEA) Report Summary described by Barlow et al. [15]. The risk rating process assesses contributors and root causes to identify remedial actions and measures of outcomes. Numerical and colour-coded ranking of potential risks and their probability are assigned (‘very high’, ‘high’, ‘medium’ and ‘low’ ratings), and the potential impacts are distilled and organised according to, in our case, previously identified functional tiers at which the risk needed to be addressed (i.e., organisational, departmental and individual) [3]. Impacts on the organisation and patients were described as negating safety, efficiency, experience and quality, all key parts of the hospital’s quality assurance goals. The reporting ratings with definitions are shown in Table 2.

## 3. Results

The results of each year’s audits, surveys and risk analyses were reviewed by members of the CoP, TH problems were identified, and, after each round of evaluations, agreed actions were taken to address prioritised problems. The 2020 findings provided a baseline for identifying the first steps towards solutions and for reporting the 2021 and 2022 results.

To illustrate the service improvement process, the following sections describe Phase 2—the problem-focused summary of audit and survey findings used to inform the initial solution design process and risk identification; Phase 3—the renewed focus on TH technology factors; and Phases 3 and 4—subsequent audit and survey results informing further risk identification and solution design. 

### 3.1. Phase Two—Problem, Risk and Solution Identification 

#### 3.1.1. Problem Identification 

To address the first two project objectives, we used information from the 2020 TH audit and confidence survey to conduct the HFMEA process. We identified ten overarching problems that could have implications or risks to the organisation, to departmental delivery of TH and/or to individual staff and patients using TH (Table 3). 

#### 3.1.2. Solution Design—Root Causes, Recommendations and Interim Solutions

Each of the factors identified in Table 3 was investigated, and their root causes were identified. The first author (the AH Service Development Officer) was tasked with developing a collated list of root causes to the identified problems and making recommendations for addressing the root causes (Table 4). The recommendations were reviewed and ratified by the AH Management Committee. 

As it was beyond the scope of the AHPs to create the change necessary in some of the identified organisational areas, those factors were set aside for advocacy activities within the organisation, but these were re-evaluated over time. Instead, we carefully selected activities that might deliver rapid results and from which we could learn and adapt future activities [16]. Allied health services implemented four practical recommendations between July 2020 and October 2020 including the following:

1. Reviewing and updating an Allied Health TH Practice Guide (with governance and instructional requirements to mitigate risks) with AH departments and CoP members.
2. Changing the TH platform and providing training across AH and administration services on how to use the new platform.
3. Review of AH and TH workstations including ergonomic set up and available equipment to support service delivery.
4. Department-led review of caseloads appropriate for TH (including reviews of evidence, benchmarking in other facilities and clinical resources to support the uptake and department-led establishment of TH key performance indicators).

### 3.2. Phases Three and Four—Review and Reassessment 

#### 3.2.1. Technology Factors

To track progress on our activities after the 2020 baseline measures, during each of the subsequent two years, we repeated the audits and surveys, checking progress against the recommendations made in Year 1. As technology problems were repeatedly raised by AHPs as the main barrier to high-quality AH TH services, we continued to include these factors in the 2021 and 2022 audits and surveys. Table 5 presents a high-level summary of our findings accompanied by the audit completion and survey response rates across the three years. 

**Table 5:** Audit completion and survey response rates for 2020, 2021 and 2022 including summary of technology-based issues (* Total AH staff head count included part-time, casual and full-time professional staff, not administration or assistant staff).

March–June 2020 (* Total AH Staff Head Count = 214)	February–March 2021 (* Total AH Staff Head Count = 204)	April–May 2022 (* Total AH Staff Head Count = 212)
Audit outcomes regarding technology factors:		
20 completed audits (32 commenced)	22 completed audits	12 completed audits (16 planned)
Two auditors (authors RP and HG)	Seven trained auditors (a Social Worker; Physiotherapist; Occupational Therapist; Speech Pathologist; Community AHP; Dietitian; author RP)	Six trained auditors (a Social Worker; Physiotherapist; Occupational Therapist; Speech Pathologist; Community AHP; author RP)
33% of staff offered a ‘technology failure plan’ to the patient at outset of TH session	36% of staff offered a ‘technology failure plan’ to the patient at outset of TH session	43% of staff offered a ‘technology failure plan’ to the patient at outset of TH session
33% of telehealth connections failed before or during the audits	With the change in TH platform, 0% of telehealth connections failed before or during the audits	25% of telehealth appointments cancelled before or during the audit (due to patient side problems, patient non-attendance). No connection dropouts recorded.
The existing troubleshooting guide was not deemed necessary in 90% of audits	With the change in TH platform, a revised troubleshooting guide was recommended	33% of staff did not have easy access to the new troubleshooting guide at TH workstations
Summary confidence survey outcomes regarding technology:		
94 responders; 69 completed staff confidence surveys (Response rate: 43.9%)	63 responders; 51 completed staff confidence surveys (Response rate: 30.8%)	36 completed staff confidence surveys (Response rate: 16.9%)
39% (n = 27) of TH users reported using TH more than 2 times/month	19.6% (n = 10) of TH users reported using TH more than 2 times/month	55% of TH users reported using TH more than 4 times/month; 33% reported using TH more than once/day
49.5% of staff confident to achieve (and troubleshoot) TH connection	62% of staff confident to achieve (and troubleshoot) TH connection	Technology use continued to be an area of low staff reported confidence (see Figure 2)

**Figure 2:**
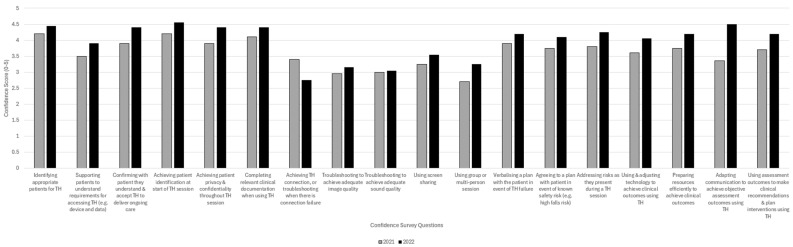
Staff confidence survey question responses for 2021 and 2022. (5–Very confident; 4–Fairly confident; 3–Neither; 2–Not very confident; 1–Not at all confident.

Information in Table 5 demonstrates a gradual improvement in factors related to technology connections, accompanied by an increase in use of TH; yet staff confidence in use of TH technology and its troubleshooting remained sub-optimal (Figure 2) as will be shown in the following section.

#### 3.2.2. Renewed Focus on TH Technology Factors

To gain a deeper understanding of the technology factors, we reviewed data in its entirety with the aim of re-examining risk factors related to organisational and clinical TH requirements. Due to the extensive amount of data gathered over three years, we present herein only descriptive data (means, numbers and percentages) to demonstrate how data were used to inform the risk rating process. 

A graphical representation of means for responses to the staff confidence survey questions repeated in years 2021 and 2022 is shown in Figure 2, demonstrating improvement in staff confidence in almost all questions. The only responses demonstrating deterioration in confidence was the ability of AH staff to achieve a TH connection or to independently troubleshoot a connection failure if it occurred at the client side. Troubleshooting ability for TH sound quality remained among the lowest ratings and largely static between years. 

**Figure 3:**
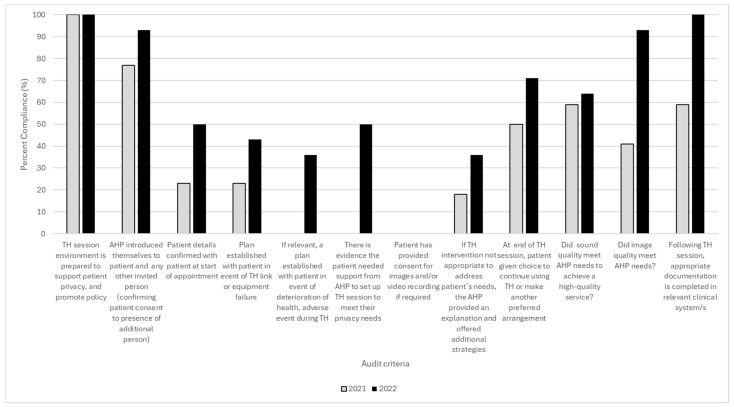
Percentage adherence by staff to best practice criteria noted during TH audits—comparison of results between 2021 and 2022 outcomes.

For audit results, an aggregated comparison of the audit criteria between 2021 and 2022 outcomes is shown in Figure 3. Most criteria improved in percentage adherence with best practice in organisational and clinical TH requirements, except for aspects related to technology problems and failure. 

To supplement the 2022 findings, a survey of AH TH patients revealed that 10 of 12 (83%) surveyed patients reported they wanted to use TH after COVID. The most common reasons patients reported for continuing to use TH included benefits of not having to travel to the hospital. Patients suggested it was important to build rapport with their therapist therefore, they preferred a mix of both face-to-face and TH appointments.

#### 3.2.3. Re-Rating of Risks

Although TH connection failures were eliminated by a change in TH platform, some technology factors were proving to be resistant to local improvement activities. Therefore, we repeated the risk rating process utilising data from each round of audits and surveys. As an example of our findings, Table 6 shows the risk ratings for 2020 and 2021, the proposed impacts of risks on health service provision and further recommendations for addressing the problems and the risks identified. The aim of each recommendation was to ensure that it was deliverable and would correct the contributing causes, with the aim of modifying risks, to strengthen allied health’s future capability to deliver TH services and their contribution to a desired state of service quality and safety. Using the HFMEA procedure, the comparison of the 2020 and 2021 risk ratings showed that seven of the ten identified performance factors shifted to a medium risk rating; however, three performance concerns did not change in rating. Those performance factors included the lack of overarching organisational TH governance, sub-optimal performance in patient identification procedure clarification when AHPs delivered TH and low AHP consensus on the appropriateness of caseload for TH. 

The process to compare audit and survey results and to re-rate risks between each year provided the opportunity to review the remedial activities and recommendations with the overall aim of achieving high-quality and safe TH practices. As a consequence of the 2020/2021 risk review process and further measurement activities in 2022, the following recommendations were made (plus some indicators of progress that have occurred since the reporting period):

1. Feedback to organisational stakeholders regarding the audit outcomes to contribute to the organisation’s digital and TH strategy. (The organisation has reviewed and changed its virtual care activities and governance).
2. Changing the TH platform to one with greater stability/reliability and the provision of training to AHPs in its use. (Improvements in confidence and capability have continued since the 2021 audits and surveys).
3. Optimising hardware resources within budget. (Dual monitors, PCs and headphones now in use at TH stations).
4. Development of a medicolegal TH responsibilities practice guide to improve staff compliance with quality and safety standards and to clarify AHP responsibility for ensuring TH devices are in working order for use in clinical care. Implementation of the revised TH practice guide is expected to mitigate risk and support AHP confidence that TH continues to be delivered correctly. (A video has been developed and is viewed by all new staff to demonstrate correct procedures for AHP use of TH).
5. To improve confidence regarding appropriate patient selection for TH, criteria of when, how and how often to use TH service delivery within AH service provision is expected to assist repositioning of TH as a first-line option in practice rather than as a stop-gap measure during crises. (Departments report monthly TH performance indicators for occasions of service and have identified specific TH caseloads, introducing patient-reported outcome measures as key features in the repositioning process).

## 4. Discussion

This paper chronicles how, using a mixed-methods service improvement approach, our organisation has responded to an increased emphasis on TH in AH practice. Our work has focused on increasing the safe and effective capability of AHPs of using high-quality TH in an acute hospital setting. We have addressed three objectives:

1. ascertaining that some organisational TH requirements were not in place at the start of the COVID-19 pandemic, yet these have been within the AH sphere of influence, and, at the time of writing, these have been addressed;
2. finding that AHP confidence to meet TH organisational and clinical requirements as determined by the survey was not always verifiable during audits of practice; hence, the mixed methods approach was justified; and
3. a co-designed TH audit tool and staff confidence survey contributed to measuring the status of AH TH capability and determining risk-associated matters that needed to be improved.

We found that improvement in TH service capability and delivery is possible in busy hospital settings, but progress towards improvement can be slow and requires continued oversight, evaluation and resourcing. Moreover, issues of technology constitute barriers to the performance of high-quality TH services in the hospital setting. Lastly, and as noted by others [16], health services are complex, and change processes are not linear. Since commencing our work, others have published information relevant to AH TH perspectives. We expect our experiences will provide guidance and shared audit/survey resources for others who may be interested in understanding the capability of health practitioners to effectively offer TH services. 

In the first phase of our work, a novel TH competency audit tool and confidence survey helped us to identify AHP TH capability and to focus on areas for improvement specific to the organisation. Enacted between May and June 2020, the audit tool assisted us in identifying if the organisational TH requirements were being delivered by AH staff. During this period, the COVID-19 pandemic created an environment within which a significant volume of ambulatory AH services was delivered mostly by interfacing with technology, resulting in significantly broader TH offerings in patient caseloads. 

To understand the staffs’ ability to achieve organisational requirements for TH, we also developed a staff confidence survey. The initial audits and confidence survey results described various and differing technology performance issues due to internal and external factors; at times, the TH platform in use was considered too unreliable by AH staff to confidently offer it as a service to the hospital’s patients. The main factor contributing to low ratings of confidence in TH technology was connection failures. Investigations into the failures led to a deeper understanding of the importance of the right technology as a facilitator of health care but also as only one part of an overall quality TH service. Problems with the reliability of technologies were progressively addressed over the period of this reporting (including a change in and training to use an alternative TH platform). As reliability issues were addressed, repeated annual audits and surveys exposed new unreported challenges such as ensuring seamless and secure linkage of TH methods with internal patient records. This factor remains unresolved with its attendant implications on patient health data privacy and security.

The reliability of technology influences factors such as ease, efficiency and comfort of TH use as we showed in Table 6, all of which are primary influencers of AHP opinions of TH suitability. Cook et al. [17] identified the same issues, and as noted earlier, health practitioner capability is associated with factors other than just knowledge and skills [4]. We pondered upon whether the appropriateness of TH caseloads and the need to meet key performance indicators may have subconsciously affected some of the broader risk indicators identified. Such factors would require further investigation. Cook et al. [17] identified other themes influencing AHPs perspectives of TH, including whether there are clear benefits of TH for consumers; whether consumers have technology access and ability; the ability by AHPs to establish and maintain effective therapeutic relationships via TH; delivering clinically appropriate and effective care via telehealth; and external influences on TH service provision. Although our work was carried out in good faith and not designed to interrogate these specific themes, we found in repeated annual evaluations an issue related to what is clinically appropriate and effective care delivery for different patient populations using TH. Cook and co-workers [17] also identified the notion of ‘assumption versus reality’ pervading their work. This theme was also evident in our work. We identified that self-reported survey confidence by AHPs of their TH capabilities did not carry over in all cases to the operationalisation of TH when practices were audited. In our earlier work [3], AHPs had made assumptions about TH access matters not supported by results reported herein. This observation supported our choice to utilise a mix of measurement methods.

During our work, we identified several non-technological issues such as clinical governance factors. Mapping these to our previous work [3], we categorised the TH evaluation issues as organisational, departmental and individual (staff and patient) tiers to optimise solution design. Responding quickly in 2020 to the changes imposed by COVID-19, we understood organisational factors required the direct involvement of senior leaders, and our first attempt at solution design would require more data to inform their future planning. Thus, our initial solutions were pragmatically designed in phase 2 to address the departmental and individual levels by clarifying best practice in a TH practice guide and reviewing TH workstations and resources alongside reviewing caseloads deemed suitable for TH. Collectively, the solutions identified in phase 2 were put in place between July 2020 and October 2020, ensuring six months of implementation before commencing re-evaluation in phase 3 in March 2021. As noted in Figure 2 and Figure 3, the activities that we implemented resulted in many improvements over time.

Over phases 3 and 4, by applying repeated audits and surveys, we interrogated how TH was being offered by AHPs in the hospital, what factors constituted potential risks or impacts on care and how we might address such matters to improve TH services. A simple example arising from the work was the early identification of ergonomic issues that were addressed through consulting with the hospital’s Workplace Safety and Quality department. The implementation of ergonomic design principles and adaptation of TH workstations enabled full body views of movement demonstrations to patients by staff in front of a computer camera. Another example was facilitating lighting location to enable improvement in identifying non-verbal cues between staff and patients. However, successful adaptive health systems in the context of digital health should consider numerous factors including not only the hardware, software and the interface between the computer and users, but also clinical content, workflows, rules and regulations and the measurement and monitoring of outcomes [18]. Our work attempted to capture as many of these factors as possible and our work will need to continue to ensure we reach our aim of offering a high-quality TH service. 

The audit and survey results have challenged the AH service to consider how best to learn from and strengthen patient-centred ambulatory care after the peak of COVID-19. Whilst departments are responsible for establishing TH caseloads, the confidence survey results showed nearly one in three staff did not initially believe their caseload was suitable for TH, improving to one in five staff (or 21%) two years later. The notion of ‘assumption versus reality’ was evident as AHPs balanced how best to interface with patients and ensure they were offered high-quality TH including the attainment of effective clinical outcomes. To address the known effect of AHP’s evaluation of TH patient suitability, we were influenced by the framework developed by Greenhalgh et al. [19] and we began to ask new questions to understand the value of TH to staff and consumers and to promote adoption. Could some patients be seen at the first appointment using TH, combined with triaging to determine the need for face-to-face follow-up? Should some patient groups be assigned to TH only? Will the skills and knowledge base of AHPs be respected for their evidence-based, decision-making abilities when determining who is afforded face-to-face treatment and which patients are assigned to health care by TH? To answer these questions, we need to better understand the benefits to consumers of delivering to them clinically appropriate and effective care. Taking this step will further evolve our TH service and more closely align it with a sustainable model of TH for AH [20].

Understanding TH competencies in the routine use of TH is vital in addressing many AH capability challenges. Anil et al. [20] reported competencies that were reflected in our capability audit criteria including communication, effective use of technology and person-centred care as well as clinical reasoning if the AH caseload is determined to be suitable. Our findings showed that AHPs reported they were more confident to use and adjust technology to achieve clinical outcomes using TH than troubleshooting technology issues when they arose. The latter issue is not likely to be addressed in the short term, whilst TH platforms cannot seamlessly be integrated across firewalls and into hospital digital infrastructure due to concerns regarding security of patient health data.

We chose to use two methods to evaluate our TH services—by capability audits and confidence surveys. This proved to be an important choice. Telehealth was considered by AHPs during audits as an appropriate method by which to continue treatment, yet only half of the respondents in the confidence surveys felt confident that their caseloads were appropriate to TH. This disjunction may have resulted from bias introduced by the nature of recruiting volunteers for the audit process. It is likely that AHPs who volunteered were more regular users of TH and had developed more mature perspectives of how TH could be used in practice. And although staff self-identified ‘Fairly’ or ‘Very’ confident levels in identifying and addressing safety and associated risks (such as achieving patient identification with TH), this was not confirmed in the results of the TH capability audits. The bias introduced into the audit process as noted above may explain these results. Yet we note the findings of Lam et al. [21] who investigated confidence in the ability by Australian health science students to master new information and communication technologies. They found that exposure to information technology training and confidence in learning new skills are related to attitudes toward engaging in e-Health. Our work offers a new insight into the domain of confidence by qualified health professionals in using TH, the importance of exposure to TH and appropriate training. It also raises questions about what level of competence is required (and in what professional and/or technology-related elements) before a health practitioner can be deemed competent to undertake TH care. 

Our work has shown that it is possible to facilitate change at various levels in an acute hospital setting albeit requiring persistence and vigilance to enact the change. Our experience shows that the identification of risks and categorising them into tiers can lead to improvements that align with frameworks for sustaining TH in AH [19]. The identification of risks can lead to their mitigation, e.g., offering interpreter services for patients with English as a second language and consent to take images or videos during TH. We believe the development, implementation and review of the AH TH practice guide is an important contributor to raising awareness and mitigating some risks. However, at the time of our third annual review in 2022, some risks remained high including the verification of patient identity and the suitability of using TH in the care of different patient populations. The identified governance factors are being addressed within the hospital with the appointment of virtual care instruments (including a committee tasked with the oversight and management responsibilities specific to TH). Awareness of clinical and technology governance of TH is expected to translate into ensuring future and emerging issues can be addressed in a timely way, e.g., threats to health data arising from the use of personal avatars [22] and artificial intelligence [23].

The audit and survey processes, although time-consuming, have proven to be valuable in providing evidence required for change processes. Our observations demonstrate that simply implementing isolated quality improvement recommendations are not enough. As confirmed by published frameworks [17,19], TH delivery of health care is not simply the transfer of an old paradigm (face-to-face treatment) to a new setting. Henry et al. [24,25] discuss the non-technical clinician attributes and interpersonal skills that may impact on the successful provision of TH. Allied health professionals are evidence-based applied clinical scientists who will respond to data. Therefore, research is required for identifying patient and resource features to be able to select the right patients and right TH parameters (e.g., frequency of TH and models of care such as TH-only or hybrid TH + face-to-face) contributing to high-quality outcomes. Our work resulted in a change to how patients can access the hospital’s AH service. Patients are now able to choose whether they receive treatment via TH or in person. Further evaluation of patient service data suggests that this opt-in approach to treatment has not adversely affected the TH use statistics, and it responds to the consumer desire for choice [26]. Whilst staff competency and training have been addressed, we highlight the importance of patient-centred services and empowering patients to understand benefits and challenges and to be supported to make choices about using TH. The need to co-design patient-facing TH resources is abundantly clear [27]. Newly added to our considerations is the need to factor patient training needs into the use of the virtual care environment, especially the patient populations less experienced in digital technologies [28].

The work described in this paper has allowed us to view TH with a broader and more learned lens. In our early work prior to COVID-19, we utilised information from the TH literature that proved to be limited for our pandemic response and future needs. For example, different patient groups and conditions, different geographic influences and models of care that primarily delivered TH with a health care professional in a remote health care setting are no longer suitable features of a high-quality AH TH service. COVID-19 has resulted in TH being delivered in the home and workplace to a broader range of patients resulting in a range of different and complex requirements to ensure the safety, confidentiality and quality of care. Underpinning all of this is the technology itself which needs to be fit for the purpose and the need to train staff in its optimal use.

Our work has become prescient due to the advent of the COVID-19 pandemic. Our work has also raised more issues that have not yet been answered. We have taken the precedent of developing and refining an audit tool and used this to continuously evaluate capability and competencies that have been raised more recently in the literature [19]. We have found an increase in staff confidence using TH, and there have been many improvements across the audit criteria. Importantly, our findings align with others in the field.

### Limitations

This service improvement and evaluation activity was not designed as a research study and is limited in a number of ways. The processes we undertook were designed to address the emerging requirements of good clinical care during a pandemic; thus, the knowledge generated is specific only to our setting. The results cannot be generalised to other settings. Our methods can be used only to guide others for developing their own procedures for tracking AHP TH capability in an acute hospital setting. The audit and survey tools were developed for our own use and have not been validated. The tools are provided as examples to others who may be interested in how we assessed staff TH competence and confidence. The evaluation of risk as described herein was based on the specific clinical circumstances at the time of each phase of work and were impacted by matters such as resourcing (e.g., staffing) and clinical timelines and workloads. Others may have evaluated the risks and responded to them in a different way.

## 5. Conclusions

We set out to understand if organisational TH requirements were being achieved when delivering AH services. We have offered our experiences to demonstrate how health service quality improvement activities are not ‘one-off’ events and how there may be many inputs into complex problems requiring potentially complex solutions over long periods. We used audits and surveys over a 3-year period to identify risks and to inform on staff needs for implementing high-quality TH services. Several risks were identified, and remedial actions resulted in improvements across most of the identified risks. And our work has influenced the hospital’s work regarding virtual care. We also wanted to understand if AHP staff were confident in meeting AH TH organisational and clinical requirements. We found that staff confidence reported in surveys did not always convert to observable competency during audits. A myriad of technology-related factors contribute to a high-quality TH service offering, and some of these factors may only be indirectly related to the technology itself. It is imperative, therefore, to triangulate service improvement activities using a mix of methods. Finally, the TH audit tool and staff confidence survey, when used as a mixed methods approach to identify risk factors served as effective tools for measuring improvement in AH TH service delivery. Work is ongoing to further mitigate risks, and new and evolving recommendations resulting from the service improvement methods we have used in our work are being used to drive further service improvements contributing to a high-quality TH service. In future, research to better understand consumer requirements of high-quality virtual health services is imperative, especially as artificial intelligence becomes more commonplace within the health and associated industries. We suggest that health professional competency standards regarding TH include not only clinical competency but some level of technology competency.

## Figures and Tables

**Table 1:** Community of Practice subsidiary questions.

Primary Objective (Question) 1	Primary Objective (Question) 2
Were the organisational telehealth requirements being achieved when delivering allied health services?	Are AHP staff confident to meet allied health telehealth organisational and clinical requirements?
Did AHPs meet informed consent, privacy and data security standards related to TH? Were access and/or equity issues identified within telehealth service provision (e.g., access to interpreter services)? Did AHPs document relevant telehealth service delivery requirements? Did AHPs check that available technology was in working order? Did technology work as it was intended for both AHP and patient?	Were AHPs able to use available technology to achieve desired outcomes? Were AHPs identifying and addressing matters related to AHP and patient safety to meet telehealth service delivery needs? Did AHPs address clinical/communication needs of clients? Did AHPs address current and ongoing acceptability and appropriateness of telehealth use?

**Table 2:** Modified Health Failure Modes, Effects Analysis Report Summary.

Severity Rating	Rating	Description/Definition
Very High Risk of task failure	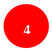	Very high probability of failure, risk and/or significant impact to organisation/patient
High Risk of serious problem	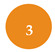	Parts of process/system demonstrate actual or potential failure with high probability of risk to organisation/patient and requiring workarounds to complete the task
Medium Risk (minor hindrance)	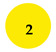	System deficits with minimal immediate impact, easily tolerated by staff/patients but with medium chance of impact to organisation/patient over time
Low Risk (no problem)	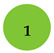	No problem or failure mode identified, process is being followed

**Table 3:** Collated problems identified from 2020 TH audits and staff confidence surveys (stratified into factors needing to be addressed at organisational, departmental or individual staff levels).

**Organisational Factors:**
- The telehealth technology access licence was not fit for large-scale use and needed to be changed to allow for high volumes of concurrent users. - There was a lack of experience at all levels of delivering high volumes of telehealth. - Hospital firewalls and wi-fi access impacted telehealth technology access using smart devices and laptops. - Governance, administrative and privacy factors were not being identified for TH practice (especially patient registration and consent when the patient is not onsite).
**Departmental factors:**
- Telehealth set up instructions for patients and clinicians to access telehealth technology were incorrect for large-scale concurrent use and needed to be changed. - Greater emphasis on telehealth IT support for clinicians and patients was required. - Quality of telehealth technology processes were not efficient resulting in a sub-optimal user experience. - There was a need for telehealth-dedicated workstations to improve lighting, ensure headset access, and access to medical notes (side-by-side computer monitors desirable to allow such access whilst observing the patient).
**Individual clinician factors:**
- In the absence of software-specific capability, novel workarounds were found, e.g., taking a screen shot (image) to capture joint range of motion. - There were significant ergonomic and fatigue issues for staff who were delivering back-to-back telehealth sessions.

**Table 4:** Root causes and recommendations to address causes.

Current State	Recommendations	Desired State
Hospital lacks telehealth governance documents.	Comprehensive telehealth governance review and establishment of hospital governance documents.	Hospital telehealth governance documents are available within hospital Document Centre.
Hospital telehealth risk mitigation is not documented and/or widely available.	Telehealth risks and appropriate mitigation strategies are identified and documented.	Risk mitigation related to delivering consumer care using telehealth at hospital is documented.
Telehealth technology/platforms used to deliver consumer services are not reliable in current setting.	Hospital to review connectivity issues identified with using telehealth in allied health.	Telehealth technology is accessible and reliable for allied health and its consumers. Telehealth experience for allied health consumers is not compromised when compared to face-to-face services.
Allied health lacks confidence in the hospital telehealth platform and technology to meet clinical service needs and is perceived to not meet consumer requirements.	Allied health to identify telehealth platform/technology requirements to achieve low-variability/high-quality clinical outcomes. Hospital to review consumer experience and requirements for telehealth.	Telehealth platforms, technology and clinical tools used to deliver allied health telehealth are efficient and effective.
Allied health clinicians lack awareness about telehealth governance requirements.	Education about hospital telehealth governance and how this should be implemented in consumer care.	Allied health demonstrate understanding of telehealth governance including risk mitigation within telehealth service delivery.
Many allied health clinicians have reduced confidence to achieve value-based care using telehealth technology/platform.	Education about using telehealth technology and platform becomes available.	Allied health is confident to use existing and new telehealth technology and platforms to achieve innovative models of care.
Many allied health clinicians have reduced confidence to achieve clinical outcomes and/or progress intervention using telehealth.	Allied health to undertake review of evidence and telehealth practices in similar services to ensure quality and safety of consumer care. Allied health to embed telehealth evidence-based practice when discussing redesigning models of care.	Allied health can effectively and confidently deliver telehealth to nominated consumer caseloads.

**Table 6:** Initial 2020 and first annual (2021) allied health staff telehealth capability risk ratings as identified using results from capability audits and confidence surveys to inform the HFMEA method with recommendations for lowering identified risks. (TH: telehealth; AH: allied health; AHP: allied health professional/s).

**1. Telehealth Governance**			
**2020** **Identified Issue or Risk**	**Severity Rating and Impacts**	**2021** **Identified Issue or Risk**	**Severity Rating/Recommendations**
Issue: Hospital lacks TH governance documents—no broad TH policy, TH guidelines or TH work instructions in the hospital Document Centre. Risk: Limited governance to guide standards of care for the consumer receiving care via TH.	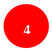 Safety Efficiency Experience Quality	Continued absence of organisational TH policy and procedural guidance.	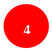 Recommendation: Escalate and advocate for greater visibility of patient identification, identifying patients if in a group TH session, of TH documentation requirements and equitable access for non-English speaking clients.
Issue: Patient identification was checked at start of TH session in only 23% of audited sessions. Risk: Significant risk when patient identification is not followed in accordance with hospital policy and available evidence-based standards for TH.	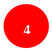 Safety Efficiency	Three forms of patient identification were checked correctly in 13/22 or 59% of observed sessions. Overall, 2 forms of patient identification were observed in 17/22 or 77% of observed sessions.	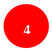 Risk: Lack of clarity in patient identification using TH. What are risks for using 2 versus 3 means of identification? Recommendation: Clarify expectations about patient identification including assumptions that a TH link and email are forms of patient identification. Clarify requirement for patient identification and if it is required for a group TH session. Advocate hospital patient identification procedure is updated to include allied health and TH.
Issue: Patient consent for images or video. Consent was obtained from the patient, for photographs or video during TH, in 0% of sessions that were audited. Auditors identified that there was no organisational governance document outlining correct process to obtain and document patient consent for photos or videos during TH. Risk: There is risk of breaching consumer privacy and consent.	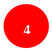 Safety Efficiency	Document audit of observed TH sessions demonstrated 8/22 or 36% had documented prior image consent. 1/22 observed audited sessions took an image during TH. Patient consent was verbally observed and documented correctly. Emerging observation: Patients are using their smart phone to take images at their side of a TH session and sharing these with allied health.	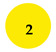 Risk: Departments do not have a mature TH orientation and training process to ensure all new staff and staff new to TH caseloads are aware of and can maintain image consent compliance. Recommendation: Establish a consistent approach to TH orientation within AH and embed the TH requirements within department caseload handovers between staff. Capture emerging technology skill opportunities to improve clinical outcomes and experiences, e.g., support patients to take images using their own mobile devices for sharing during TH.
Issue: TH should be accessible to all hospital patients, including patients with English as a second language. For the patient who speaks English as a second language, there was documentation that an interpreter was offered in 0% of the audited TH sessions. Family or no qualified interpreter was used. Risk: If patient not being asked if they need an interpreter, there is a risk that their health care needs will not be met.	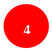 Safety Efficiency Experience Quality	100% compliance	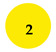 RISK: Hospital policy for engaging interpreting services should be reviewed to clearly include how to support patients with making an informed decision about accessing TH. Currently, patients of allied health may be verbally offered TH over the phone creating health literacy barriers to accessing TH. Recommendation: Discuss how to minimise health literacy barriers for non-English speaking patients to effectively access TH.
Issue: TH technology or connection failure risk management. During observed audits, AHPs were observed making a plan with the patient in case of technology failure in only 23% of sessions. Risk: Planning for the event of TH technology or connection failure is recommended within evidence-based guidelines and an important process for risk mitigation.	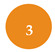 Safety Efficiency Experience Quality	36% of observed sessions completed technology failure planning. Incidence of failure to connect observed in 0% of audits.	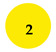 Risk: Currently very low TH failure including connection or dropping out; however, when an issue occurs only 1 in 3 patients were provided with a plan to ensure efficient re-establishment of communication. Potential impact to client experience and allied health confidence delivering TH service. Recommendation: Achieve standardised allied health technology failure patient instructions. Consider embedding instructions within email appointment letter/patient TH brochures. Ask each department to ensure troubleshooting tips are visible and beside each TH station.
Issue: Patient safety risk management. During observed audits, AHPs were observed making a plan with the patient in the event of patient deterioration in only 14% of sessions. During the audit, 0% of the AHPs identified a change to patient safety and/or took steps to mitigate or manage the change. Risk: Planning for the event of patient deterioration/change in patient safety is recommended within evidence-based guidelines and an important process for risk mitigation.	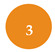 Safety Efficiency Experience Quality	During the audit 5% or 1/22 observed sessions put a plan in place to manage patient deterioration; and 13.6% of AHPs identified a change to patient safety and took steps to mitigate the change.	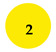 Risk: Departments do not have a mature TH orientation and training process to ensure all new staff and staff new to TH caseloads maintain compliance with patient safety during TH encounters. Recommendation: Establish a consistent approach to TH orientation within AH and embed TH requirements within department caseload handovers.
**2. Telehealth technology**			
**2020 ** **Identified issue/risk**	**Severity rating and impacts**	**2021** **Identified issue/risk**	**Severity rating/recommendations**
Issue: TH connection failures. During the audit 38% of TH sessions experienced a connection failure. 90% of AH clinicians who completed the survey used the hospital recommended TH platform. Risk: When there is a connection failure, there is risk that the patient will not receive safe, efficient quality care and this will negatively affect the patients’ experience and clinical outcomes and staff confidence in the use of TH. (This theme identified also in the staff confidence survey.)	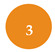 Safety Efficiency Experience Quality	During audits, 0% of allied health was unable to connect; 2 patients experienced connection issues from patient side. 100% TH sessions observed in audit used newly recommended hospital TH platform. Survey responses demonstrated 76% predominantly used new TH platform since September 2020. Survey responses demonstrated 62% felt confident to connect and troubleshoot connection failures.	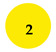 Risk: Disconnect between audit observed issues with TH connection and overall reported survey confidence to connect and troubleshoot connection failure. Complacency (or fatigue) related to acceptance of TH issues rather than reporting to drive improvement. When there is no troubleshooting capability in place for TH failure, any problem must be escalated to find solutions for quality service provision. Recommendation: Investigate and establish a simple method to log issues with TH manager to assist with a continuous improvement approach to building patient instructions or staff knowledge about TH.
Issue: TH image and sound quality. During the audit, 41% of sessions were observed to have reduced quality of sound and 61% reduced quality of image. Allied health clinicians accepted the sub-optimal quality during TH sessions. Risk: When there is less than optimal quality of sound and/or image during a TH session, there is risk that the patient will not receive safe, efficient quality care negatively impacting the patient’s experience and clinical outcomes. There is also the risk that the AHPs will need to compensate for low-quality sound and image leading to fatigue and reduced confidence in using TH.	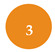 Safety Efficiency Experience Quality	During the re-audit <1% sound issues and 13% image issues observed. AH TH confidence survey reported 47% of AHPs feel confident to troubleshoot sound and image problems. TH was converted to phone calls on 2 occasions due to client-side issues with camera.	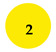 Risk: AHP confidence to troubleshoot for image and sound quality demonstrates little change in confidence despite TH training and hospital IT support. Low reported frequency of TH service delivery suggests AHPs lack practice. Recommendation: Update and republish TH “how to use” instructions including troubleshooting quick guide for AHPs. Review and update troubleshooting expectations using new TH platform. Define what issues should be reported to hospital digital technology services. Consider TH scenario problem-solving opportunities to build confidence.
Issue: Lack of ergonomic TH workstations. Audit and survey results showed that AHPs did not have consistent access to an ergonomically appropriate TH workstation. Allied Health clinicians used a tablet or laptop in 55% of audited sessions. Risk: If AHPs are not provided access to a TH workstation that meets the safe ergonomic hospital workplace health and safety recommendations, there is potential risk to clinician health.	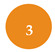 Safety Efficiency Experience Quality	Audit observations: 73% of sessions used desktop PC or laptop with access to TH dedicated devices/workstations. Audit observations: 23% of AHPs used tablets. Auditors observed staff with sub-optimal posture when using tablets. Audit observations demonstrated lighting, noise and privacy was 100% effective. AH confidence survey feedback included requests for more TH dedicated spaces including improved lighting in front of AHPs and having access to 2 monitors and headsets and microphones.	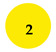 Risk: Excellence in delivering TH is compromised with poor access to required resources. TH workstation expectations by AHPs had evolved with more experience of TH in previous 12 months. Some TH workstations remain technologically inefficient challenging efforts to use TH. Recommendation: Achieve minimal standards for a TH workstation including access to 2 screens, head set and microphone. Workarounds (e.g., tablets) must support ergonomics. Further investigate solutions to support efficiency and clinical outcomes.
**3. Allied health professional support**			
**2020** **Identified issue/risk**	**Severity rating and impacts**	**2021** **Identified issue/risk**	**Severity rating/recommendations**
Issue: Appropriateness of AH patient caseloads for TH. The survey demonstrated that 40% of AHPs believe their caseload was appropriate for TH. Risk: If incorrect patient caseloads are selected for TH, it may be difficult for the AHPs to provide efficient, safe care with positive clinical patient outcomes.	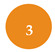 Safety Efficiency Experience Quality	AHP survey revealed 46.4% of responders said their caseload was appropriate for TH. AHP survey reported concerns about impact of TH key performance indicators on patient selection for TH services.	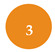 Risk: Mismatch between AHP belief about patient suitability for TH, and department-led patient TH caseload selection. Risk: Lack of data from patients and lack of evidence from literature regarding selection of appropriate TH caseloads. Recommendation: Patient feedback from TH caseloads to provide guidance to AH departments on TH caseload suitability. Departments to continue to review best practice and performance indicators for each of their nominated TH caseloads.

## Data Availability

Original contributions presented in the work are included in the article and Appendix A. Further inquiries regarding data can be directed to the corresponding author/s.

## Appendix A

**Table A1:** Allied health telehealth audit criteria.

Audit Questions	Observation
Consent obtained from allied health clinician (to participate in audit)	Yes/No
Consent obtained from patient (for audit)	Yes/No
Is this the first telehealth session for the patient?	Yes/No
Is this the first time the allied health clinician has undertaken telehealth with this patient?	Yes/No
The patient has informed consent documented in <hospital patient management system>	Yes/No
The patient has provided consent for images and/or video recording if required	Yes/No
Following the telehealth session, appropriate documentation is completed in the relevant clinical system/s (Note: including progress notes for telehealth session, noting patient consent for treatment and patient consent for audit)	Yes/No
A hazard or incident identified within documentation for the telehealth session is also recorded in <appropriate hospital system/s>	Yes/No/Not Applicable (NA)
Is English the patient’s primary language?	Yes/No
Where English is a second language for the patient who has accepted telehealth, there is documentation an interpreter has been offered and or planned/available for the telehealth appointment	Yes/No/NA
There is evidence that allied health have set up and tested the patient’s telehealth session link, microphone and camera, prior to connecting with the patient in a telehealth session	Yes/No
The environment where the telehealth session is being conducted is prepared to support patient privacy, and promote professional <hospital> brand including uniform policy Environment lighting is prepared and used effectively to support telehealth	Yes/No
Environment noise is managed prior to starting the telehealth session	Yes/No
Camera set up is prepared prior to the telehealth session	Yes/No
In the situation that allied health equipment was not working, there was support to troubleshoot the issue and not interfere with the scheduled appointment	Yes/No/NA
The allied health clinician introduced themselves to the patient and to any other invited person (confirming that the patient agrees to the presence of the additional person)	Yes/No
The patient’s details were confirmed with the patient at the commencement of the appointment	Yes/No
A plan was established with the patient in the event of telehealth link or equipment failure, e.g., patient’s phone number is confirmed	Yes/No
If relevant, a plan was established with the patient in the event of deterioration of health, adverse event, e.g., agree to call ambulance	Yes/No/NA
When a change to patient safety was encountered, the allied health clinician recognised this and took steps to mitigate or manage it	Yes/No/NA
When a change in privacy risks was encountered, the allied health clinician recognised this and took steps to respect and protect the patient’s privacy, including ending the call at the end of the session	Yes/No/NA
<Hospital’s> professional image was maintained through adhering to internal standards of professional conduct	Yes/No
During the telehealth session, the sound quality did not meet clinical expectations, e.g., broken sound, delayed sound, one person could not hear properly, etc.	Yes/No
During the telehealth session, the picture quality did not meet expectations, e.g., pixelated, blurry, temporary issues	Yes/No
The telehealth link experienced a connectivity failure before or during the session and the patient was contacted by phone	Yes (before)/Yes (during)/No
Which device was used in the telehealth session?	Desktop/Laptop/Tablet/Other
Was a photo or video taken during the telehealth session?	Yes/No
Was screen sharing used during the telehealth session?	Yes/No
Was this a group session?	Yes/No
There is evidence the patient did not test the telehealth session link and microphone and camera prior to connecting to the telehealth session	Yes/No
There is evidence the patient needed support from the allied health clinician to set up the telehealth session in a quiet and well-lit environment	Yes/No
The allied health clinician supported the patient to set up the telehealth session in a quiet and well-lit environment	Yes/No
There is evidence the patient needed support from the allied health clinician to set up the camera angle to meet telehealth session needs	Yes/No
The allied health clinician supported the patient to set up the camera angle to meet telehealth session needs	Yes/No
There is evidence the patient needed support from the allied health clinician to set up the telehealth session to meet their privacy needs	Yes/No
The allied health clinician supported the patient to set up the environment to meet privacy needs	Yes/No
There is evidence the patient needed support from the allied health clinician to meet space requirements for the telehealth session	Yes/No
There is evidence the allied health clinician provided the patient with an opportunity to give feedback/an update about their allied health care needs	Yes/No
If telehealth intervention was not appropriate to address the patient’s needs, the allied health clinician provided an explanation and offered additional strategies	Yes/No/NA
At the end of the telehealth session, the patient was given the choice to continue using telehealth or make another preferred arrangement	Yes/No/NA

**Table A2:** Questions included in the survey of allied health staff confidence.

Q1:	Have you used telehealth in the last 3 months?					
Q2:	What platform do you usually use for patient telehealth services? Can select more than one.					
	Outpatient telehealth session using Vidyo		Outpatient telehealth session using Lifesize		Inpatient telehealth session, e.g., Teams	
Q3:	Describe how frequently you have delivered telehealth sessions in the last 3 months:					
	More than one session/workday	One session/workday	One session/week	One session/2 weeks	One session/month	Less than one session/month
Thinking about broader organisational requirements and processes, answer the questions below using the following rating scale: Very confident Fairly confident Neither Not very confident Not at all confident						
Q4:	In the next questions, please indicate what your Confidence level is to meet the organisational requirements and processes with telehealth					
	Identify appropriate patients for TH	Support patients to understand what the requirements are to access TH, e.g., device, adequate data	Confirm with the patient that they understand and accept TH, to deliver ongoing care	Obtain and document informed consent by patients for TH	Achieve patient privacy and confidentiality during TH	Complete documentation related to aspects of clinical care when using TH
Q5:	Following on from the previous question, do you have any further suggestions to improve processes to achieve organisational requirement using TH?					
Q6:	Do you believe that your primary clinical caseload is appropriate for TH?					
Q7:	In the next questions, please indicate what your confidence level is to use the TH technology:					
	Achieve TH connection or troubleshoot when there is a connection failure	Troubleshoot to achieve adequate image quality	Troubleshoot to achieve adequate sound quality in session	Use screen sharing	Use whiteboard	Group or multi-person sessions
In the next questions, please think about your recent experiences delivering TH:						
Q8:	Are you confident to identify and address patient safety risks within the TH service, this includes:					
	Verbalise a plan with the patient in case of a TH failure		Agree to a plan with the patient, in the case of a known safety risk,, e.g., high falls risk		Addressing risks as they present during a TH session	
Q9:	Are you confident to achieve clinical outcomes using TH, this includes:					
	Use and adjust technology to achieve clinical outcomes using TH		Prepare resources efficiently to achieve clinical outcomes	Adapt communication to achieve clinical understanding and develop patient clinician relationship using TH	Adapt communication to achieve objective assessment outcomes using TH	
	Use assessment outcomes to make clinical recommendations and plan interventions using TH		Achieve progress between TH sessions	Achieve clinical outcomes using TH, that satisfy patient clinical expectations	Support allied health students to achieve clinical outcomes through TH	
Q10:	Do you have any suggestions on how we can improve processes to achieve positive clinical outcomes with patients, using TH?					
Q11:	Do you have any comments or suggestions that you would like to share before exiting the survey?					
